# Influence of the Ripeness Stages of the Precursors on the Optical Characteristics of Carbon Dots Obtained from Valencia Orange Peels (*Citrus sinensis* L. Osbeck) by Hydrothermal Synthesis

**DOI:** 10.3390/nano16120783

**Published:** 2026-06-22

**Authors:** Juan Pablo Ocampo-Arias, Ángela J. García-Salcedo, Liliana Tirado-Mejía

**Affiliations:** Grupo de Optoelectrónica, Instituto Interdisciplinario de las Ciencias, Universidad del Quindío, Cra 15 Calle 12N, Armenia 630002, Colombia; ajgarcia@uniquindio.edu.co (Á.J.G.-S.); litirado@uniquindio.edu.co (L.T.-M.)

**Keywords:** ripe precursor, unripe precursor, self-doped CDs, fluorescent CDs

## Abstract

The composition of the surface, optical response, and size of the carbon dots synthesized from Valencia orange peels (*Citrus sinensis* L. Osbeck) were studied. The peels used in the hydrothermal synthesis were at three ripeness stages, and the synthesis was carried out at 220 °C and 3 MPa. Infrared spectroscopy results showed that carbon dots synthesized from the peels of unripe oranges are functionalized with oxygenated groups, and the carbonization process was effective. Instead, carbon dots obtained from peels of ripe oranges exhibit a nitrogen-functionalized surface. These results were confirmed by the bond-breakdown analysis in photoelectron spectroscopy. Additionally, the self-doped surface modified the optical response of the carbon dots, exhibiting an enhancement of the absorption band located at 283 nm corresponding to the contribution from n-π* transitions in nitrogen. Also, the excitation and emission wavelengths present a red shift for the ripe peels. Based on the above and the transmission electron microscopy results, it is concluded that the emission mechanism is associated with surface states and not particle size. Statistical analysis yielded an average size of less than 10 nm, regardless of the orange peels’ ripeness stage. It was observed that the CDs-N3 sample has more crystalline nuclei, which is justified because ripe peels follow a shorter carbonization pathway.

## 1. Introduction

Carbon dots (CDs) are nanostructures of carbon with interesting optical properties, which make them useful as biomarkers and biosensors due to their high light emission and fluorescence [1,2]. The luminescence properties of this nanomaterial are attributed to the functionalized surface, which depends directly on the precursor material used to obtain the carbon dots. This determines the chemical composition and the functional groups present on the surface, responsible for the optical emission [3]. Carbon dots were first described by Xu et al. in 2004 [4], and since then, they have attracted considerable interest due to their biocompatibility, which has contributed to the development of low-cost, low-impact environmental markers [5]. In general, carbon dots can be obtained from chemical reagents as well as from organic matter [6], and the synthesis methods are classified as top-down and bottom-up. In the former, graphite or carbon-based structures are fragmented, and from these parts, carbon dots are synthesized. The second approach uses carbon-rich organic material as precursors and carbonizes them, thereby obtaining CDs with surface functional groups derived from the precursor’s chemical components [6], which are mostly plant-based matrices. There are also various methods for synthesizing CDs, among which the hydrothermal method stands out. Since water is used as the solvent for the hydrolysis of the structures and for the subsequent formation of functionalized dots, the procedure is non-toxic. Furthermore, it is a simple, economical, and environmentally friendly technique; it allows control of the reaction conditions, thus favoring the production of highly stable CDs and good dispersion in aqueous media [7].

When carbon dots are synthesized from biomass, their optical properties depend on the chemical characteristics of the precursors [8]. In particular, orange peels are agro-industrial waste from orange cultivation and can be produced at various stages of the production chain, such as during mechanical harvesting of unripe fruit, during rejection due to underdeveloped sugars, and after juice extraction from ripe fruit. Consequently, not all industrial waste is at the same stage of ripeness. Therefore, when using vegetable matrix waste, it is necessary to consider the ripeness stages, understanding this process as a series of reactions that transform the green fruit into yellow through the degradation of chlorophyll and the synthesis of carotenoids, and from hard and acidic states to soft and sweet ones through the hydrolysis of structural polysaccharides (cellulose, hemicellulose, and lignin) into simple sugars and the metabolism of organic acids. Similarly, proteins are broken down into simple amino acids. Thus, the composition of these compounds increases or decreases depending on the stage of ripeness [9].

Various studies have been conducted on the hydrothermal synthesis of carbon dots from plant materials (fruit peels, fruit juices, plant stems, crop residues, etc.) [10], thus contributing to solutions to the problem of organic waste management. Although there are extensive reports on the changes in the dot characteristics as a function of synthesis parameters [11], analyses have not yet focused on the structural changes in carbon dots when the precursor’s ripeness stage changes.

It is well established that organic acids, particularly citric acid, accumulate during the early stages of fruit development and decrease progressively during ripening due to metabolic consumption [12]. This reduction is associated with its role as an intermediate in the tricarboxylic acid cycle, where it is enzymatically degraded and utilized as a respiratory substrate, leading to lower acidity in ripe fruits [13]. Therefore, variations in the maturity stage of orange peels are expected to influence their chemical composition, which may directly affect the carbonization process and the surface functionalization of the resulting carbon dots.

It is noteworthy that carbon dots with different surface groups are obtained using a precursor of the same species, and that this modulates the optical response. This is the case for orange peels, in which the higher citric acid content enhances the efficiency of the carbonization process in unripe peels. Furthermore, the presence of heteroatoms arising from the precursor’s composition has been identified as a key factor in modulating both the emission wavelength and the photoluminescence quantum yield. These results underscore the need for further research from both an economic and green-synthesis perspective to determine the optimal synthesis parameters and separation processes for obtaining self-doped carbon spots.

## 2. Materials and Methods

### 2.1. Precursor’s Preparation

Valencia oranges (*Citrus sinensis* L. Osbeck) were acquired from the local market and selected according to the colorimetric table (NTC 4086), in which color is designated by a number and is associated with a ripeness stage. Three ripeness stages were chosen: 0, 3, and 6, with 0 being the unripe stage. In this work, we named them N1, N2, and N3, with N1 being the unripe stage. The fruits were washed, disinfected, and then peeled. The peels were dried at 60 °C for 72 h in a convection oven. Since all samples were subjected to identical drying conditions, any thermal effects are considered uniform and do not bias the comparative analysis. The dried material was then pulverized and sieved through a 106 μm mesh. The powder was packaged in polyethylene bags for storage at room temperature until later use.

### 2.2. Carbon Dots Synthesis

**Figure 1:**
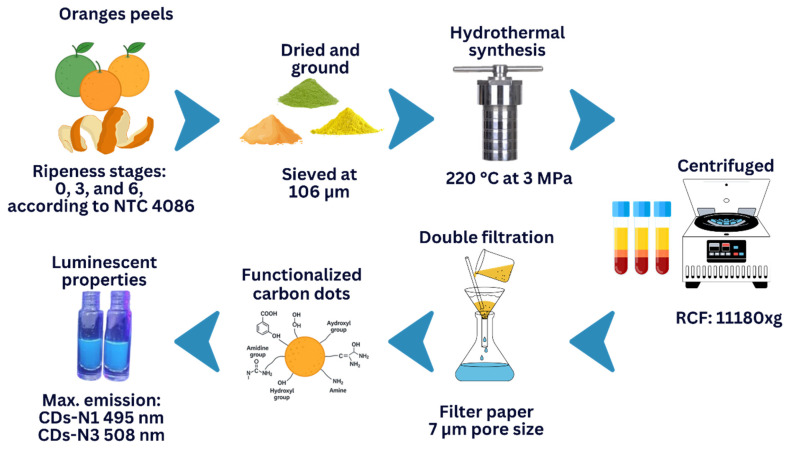
Description of the carbon dots synthesis by hydrothermal treatment from orange peels.

The CDs were obtained by hydrothermal synthesis from Valencia orange peels at three ripeness stages. The powdered precursor was dissolved in ultrapure water at a ratio of 6.75 g/100 mL and stirred continuously for 1 h at 80 °C. This mixture, upon cooling to room temperature, was centrifuged at 4025× *g* for 20 min. The resulting supernatant was subjected to hydrothermal treatment under controlled temperature and pressure. After this procedure, the mixture was centrifuged at 11,180× *g*, and the resulting supernatant contained the carbon dots diluted in water. This procedure is described in Figure 1.

The hydrothermal synthesis parameters were 220 °C and 3 MPa, selected from a previous study in which temperatures and pressures were varied across the three maturity stages (0, 3, 6), as these conditions yielded luminescent emission and nanometric particle sizes.

### 2.3. CDs Characterization

The functional groups on the surfaces of the synthesized carbon dots, as well as on the precursor powders, were characterized using mid-infrared absorption with an FTIR JASCO 4700 spectrometer (JASCO, Tokyo, Japan) equipped with an ATR accessory with a diamond crystal guide. To determine the main chemical bonds of the nanoparticles, X-ray photoelectron spectroscopy (XPS) was used as a complementary technique. The system is a Specs near-ambient-pressure X-ray photoelectron spectroscopy (NAP-XPS) with a PHOIBOS 150 1D-DLD analyzer (SPECS, Berlin, Germany) and a monochromatic Al-Kα X-ray source (1486.7 eV) with a step energy of 85.6 eV for the general spectra and 20 eV for the high-resolution spectra. The analysis of the core structure of the nanoparticles was performed in a HORIBA XploRA PLUS Raman spectrometer (HORIBA SAS, France) with a laser excitation of 785 nm and a diffraction grating of 600 gr/mm. The absorption response in the UV-vis range, which provides information on transition energies between molecular orbitals, was obtained using a standard quartz cell with an Agilent Technologies 8453 UV-visible spectrophotometer (Agilent Technologies Inc., Santa Clara, CA, USA), with tungsten and deuterium lamps. Additionally, the optical emission response to different excitation wavelengths was measured using a Horiba Jobin Yvon FluoroMax Plus spectrofluorometer (HORIBA Scientific, Kyoto, Japan), with a xenon lamp as the source, in a quartz cell with a 10 mm optical path length. The morphology of the CDs was observed using a JEOL ARM200F high-resolution transmission electron microscope (HR-TEM) (JEOL Ltd., Tokyo, Japan) operated at 200 kV, obtaining high-magnification micrographs (40,000× and 1,200,000×).

## 3. Results and Discussion

### 3.1. FTIR Absorption Spectroscopy

**Figure 2:**
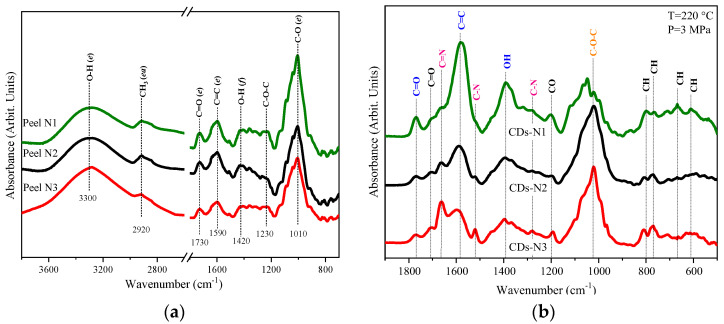
Mid-infrared absorption spectra of (**a**) dry powder obtained from Valencia orange peels. The dashed lines indicate the wavenumbers of the main bands and the assigned functional groups. The letters in parentheses represent stretching (*e*), asymmetric stretching (*ea*), and bending (*f*). (**b**) CDs synthesized at 220 °C and 3 MPa, at three ripeness stages, from Valencia orange peels.

The influence of the precursor on the surface characteristics of the CDs was analyzed by comparing the functional groups formed from the precursor at three ripeness stages. The main functional groups of the precursors are shown in Figure 2a. The band centered at 3300 cm^−1^ corresponds to the O–H stretching of the hydroxyl group and intensifies with the ripeness of the precursor due to the increase in the content of reducing sugars and compounds with free hydroxyl groups derived from structural polysaccharides (cellulose, hemicellulose, and lignin) degradation during ripening [14]; the band at 2920 cm^−1^ corresponds to the asymmetric CH_3_ stretching; the band at 1730 cm^−1^ corresponds to the hemicellulose band of the C=O stretching of the carbonyl group [15]; the band around 1590 cm^−1^ is attributed to the C=C stretching of aromatic rings present in phenolic compounds and lignin, and is more pronounced in the green peels, indicating a higher content of aromatic structures associated with phenolic metabolites characteristic of unripe plant matrices [16]; the band around 1420 cm^−1^ is assigned to O–H bending; the band at 1230 cm^−1^ corresponds to the symmetric stretching vibration of the C–O–C group (lignin band) [15]; the band centered around 1010 cm^−1^ is assigned to the stretching of the C–O bond in primary alcohols (–CH_2_–OH/–CH–OH groups) present in the plant matrix; the decrease in intensity in this band with advancing maturity indicates a reduction in the content of these free alcohols or the polysaccharides that contain them [17]. Although the FTIR spectra of the precursors are very similar to each other due to the predominant response of the polysaccharides, these spectra allow us to explain the decrease in cellulose and the increase in polyol species produced during the ripening process.

On the other hand, comparing the FTIR spectra of the carbon dots synthesized from the peels at three ripeness stages (Figure 2b) reveals a marked influence of the maturation stage on the composition of the CDs. These spectra tell the story of the reactions that occurred during the hydrothermal process. The main differences are observed in seven bands, whose relative intensities change as the maturation process progresses.

The spectra of CDs from unripe peels suggest a different carbonization mechanism than that observed in the other two states. For example, the band at 1020 cm^−1^, corresponding to C–O–C vibrations, is significantly attenuated, indicating greater depolymerization of structural polysaccharides in this biomass. This process is favored because unripe peels contain higher levels of citric acid, which acts as a catalyst in the hydrolysis reaction. Consequently, an increase in the relative intensities of the bands labeled C=O, C=C, and OH is observed, reflecting the condensation and polymerization of chemical species fragmented during monomer dehydration reactions to form CDs. The 1580 cm^−1^ band of the C=C bonds increases due to the presence of more aromatic groups, while a more oxygenated surface is observed in the stretching vibrational modes of the C=O at 1780 cm^−1^ and OH at 1390 cm^−1^. This FTIR response is characteristic of carbon dots with oxygenated surfaces [18]. The mentioned reactions are also favored by the presence of citric acid, as it is well known to participate in all stages of hydrothermal carbonization, maintaining an acidic environment conducive to nucleophilic attack, increasing nucleation by modulating graphitization, and contributing oxygenated groups to the surface [19].

On the other hand, the CD spectra from intermediate (N2) and mature (N3) peels suggest that the carbonization mechanism is primarily driven by free sugars from natural ripening, since no cellulose depolymerization is observed (the C-O-C band does not decrease). This behavior is associated with the gradual decrease in citric acid in the fruit, which discourages hydrolysis reactions, causing incomplete carbonization of the precursor material and, consequently, no increase in the C=C band, indicating reduced formation of aromatic groups.

It is also observed that, as ripeness increases, bands emerge at 1660 cm^−1^, 1518 cm^−1^, and 1278 cm^−1^, associated with the C=N stretching vibrations of imines (the first band) and the C-N vibrations of amides (the other two). This indicates that CDs with surface nitrogen are obtained from mature peels [20,21].

It is worth noting that proximal analysis of the precursor materials revealed a slight increase in crude protein content on a dry matter basis with ripeness stage, from 7.58% in N1 to 8.06% in N3. Therefore, nitrogen incorporation into the CDs is not associated with this availability, but rather with the preferred, lower-energy pathway, which, in this case, begins with the dehydration of free monomers. These monomers then condense with amino acids during polymerization so that the nitrogen can end up in both the core and the surface. Furthermore, the bands at 800 cm^−1^ and 760 cm^−1^, corresponding to the C–H bonds of the aromatic groups, are more intense, indicating increased graphitization [18].

These results indicate that the carbonization process is more efficient with unripe biomass due to its higher citric acid content; furthermore, the resulting carbon dots exhibit a less graphitic core and a surface functionalized with oxygenated groups. Although carbonization with ripe biomass is incomplete, it produces more graphitized carbon dots with a nitrogen-functionalized surface.

### 3.2. X-Ray Photoelectron Spectroscopy

**Figure 3:**
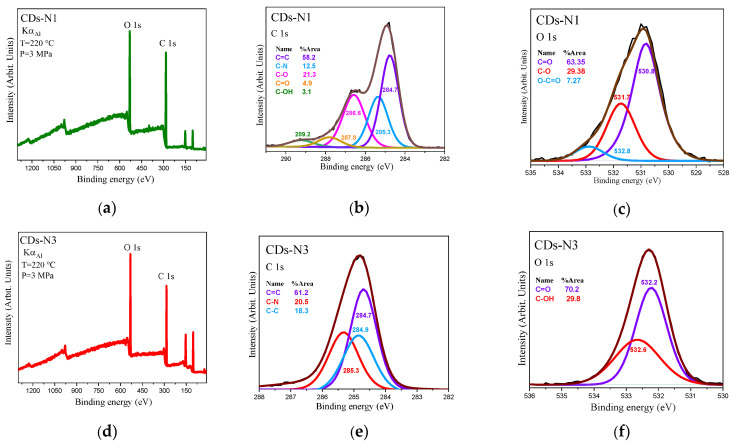
XPS spectra for CDs-N1 (**a**) survey range, (**b**,**c**) high resolution for carbon and for oxygen; for CDs-N3 (**d**) survey range, (**e**,**f**) high resolution for carbon and for oxygen.

The surface chemical composition and bond states of the carbon dots obtained from orange peels were evaluated using XPS, where CDs-N1 were synthesized from unripe peels and CDs-N3 from ripe peels. XPS analysis was performed on representative samples corresponding to the extreme ripeness stages (N1 and N3) to highlight the most significant differences in surface chemistry. Figure 3 shows the survey spectra, in which the predominant response is due to carbon and oxygen. The contributions of silicon from the substrate on which the samples were deposited are ignored. The identification of the C and O bond states was performed by graphical analysis of the contributions to the high-resolution spectrum.

The spectra showed dominant signals at 284.8 eV (C 1s) and 531.8 eV (O 1s), confirming the formation of carbonaceous materials with a predominantly oxygenated surface. In both samples, the high-resolution XPS spectra of carbon revealed contributions associated with sp^2^/sp^3^ hybridizations (C–C/C=C), along with C–O, C=O, and O–C=O bonds, indicating the coexistence of carbon-rich regions and a high density of oxygenated functional groups generated during the synthesis of the precursors [18]. Analysis of the high-resolution C 1s spectra (Figure 3b,e) revealed differences associated with the chemical environments. The percentage contribution, calculated from the area under the curve, shows that the C-N bond has a higher contribution to the spectrum in sample CDs-N3 than in sample CDs-N1, confirming the predominance of nitrogen on the surface of the carbon spot and explaining why, in this sample, nitrogen is clearly visible in the FTIR spectra.

Likewise, the high-resolution spectra (Figure 3e,f) confirm that CDs-N3 has less variety of carbon-oxygen configurations. This result suggests greater heterogeneity of oxygen-bonded functional groups in the sample obtained from unripe peels (CDs-N1) compared to the samples obtained from ripe peels (CDs-N3), although the latter is primarily decorated by nitrogen.

### 3.3. Raman Spectroscopy Analysis

**Figure 4:**
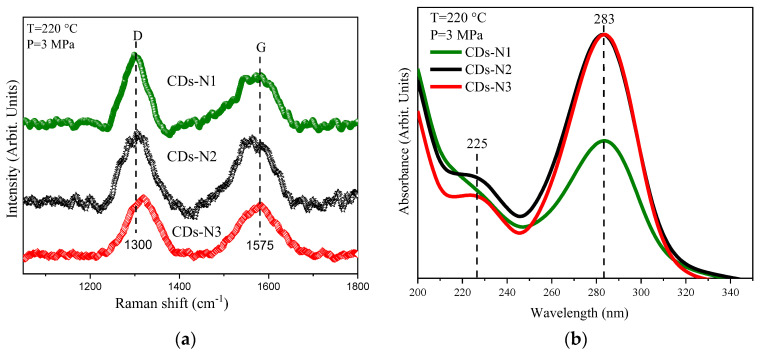
Raman spectra (**a**) and UV-Vis absorption spectra (**b**) of CDs synthesized at 220 °C and 3 MPa, obtained from Valencia orange peel in three ripeness stages.

Figure 4a shows the similarity among the Raman spectra of CDs synthesized from orange peels at three ripeness stages. The spectra exhibit two broad bands, characteristic of graphitic bonds, centered at 1300 cm^−1^ and 1575 cm^−1^, corresponding to the D and G bands, respectively.

As widely described in the literature for biomass-derived carbon points, the D band corresponds to sp^3^-hybridized carbon vibrations associated with structural disorder and defects, while the G band reflects C=C bond-stretching vibrations in sp^2^-hybridized carbon regions related to graphitic ordering [22]. A gradual attenuation of the D band is observed from CDs-N1 to CDs-N3, which could indicate greater relative structural order. The broadening of both peaks across all samples reflects the presence of heteroatom-doped domains and amorphous regions typical of hydrothermally treated biomass, where incomplete graphitization and local structural disorder inhibit the development of well-defined phonon modes [23].

### 3.4. UV-Vis Absorption Spectra

The UV-Vis absorption spectra of the synthesized CDs show two bands (Figure 4b): a low-intensity one around 225 nm and a more pronounced one around 283 nm. The first band is attributed to π–π* transitions of aromatic C=C bonds within the sp^2^ conjugated domains of the carbon core, indicating the presence of aromatic structures in the carbon dots. However, this band alone cannot be used as a direct indicator of the material’s degree of graphitization. The second, more intense band corresponds to n–π* transitions associated with carbonyl (C=O) groups and other surface functional groups containing heteroatoms, such as amides and N–H bonds, present on the surface of the CDs [11].

In the UV-Vis absorption spectra of the peel powder, no differences were observed between the ripeness stages. Compared to those of the CD samples, the spectra were less intense and exhibited less defined absorption bands. This suggests the presence of well-defined molecular orbitals or levels in the CD samples, that is, more ordered electronic structures after synthesis. In particular, the increased intensity of the band around 283 nm in the more ripe samples should not be interpreted solely as increased graphitization, but may be related to a greater contribution from electronic transitions associated with surface nitrogen groups. This behavior agrees with the FTIR results, which show an increase in the bands associated with N-H vibrations and amide modes (1660 and 1520 cm^−1^) [24].

### 3.5. Fluorescence of CDs

**Figure 5:**
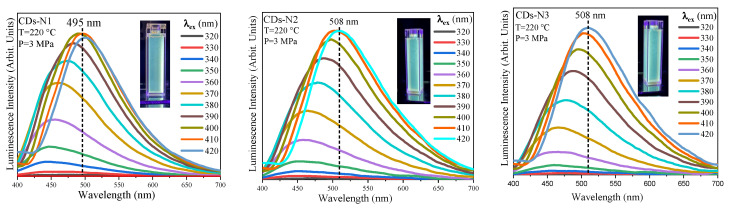
Emission spectra of CDs synthesized at 220 °C and 3 MPa, obtained from Valencia orange peels at three ripeness stages.

The spectra in Figure 5 show the emission behavior of CDs synthesized from orange peel at different ripeness stages. When excited at wavelengths between 320 nm and 420 nm, the CDs exhibit varying positions of maximum emission, indicating a dependence on excitation wavelength and suggesting differences in core structure and surface states among the samples. Sample CDs-N1 shows an emission maximum at 495 nm for an excitation of 410 nm, while CDs-N2 and CDs-N3 show shifts in the maximum towards 508 nm with an excitation of 420 nm. When the CDs are oxygenated (CDs-N1), oxygen acts as an n-type dopant for carbon; the levels generated are deep, meaning that the energy required for the transitions is higher than in the case of nitrogen as a dopant.

The red shift in the emission maximum observed with increasing precursor ripeness (Cds-N3) can be attributed primarily to the incorporation of nitrogen functional groups on the CDs’ surface, rather than to an increase in the degree of graphitization of the carbonaceous core. In particular, the presence of N–H bonds and amide-like groups, identified in the FTIR spectra of samples derived from more ripe peels, can introduce new surface electronic states that act as lower-energy emitting centers. This behavior has been reported in the literature for nitrogen-doped carbon dots, where the redshift is related to modifications in the surface electronic structure rather than to an increase in the size of sp^2^ domains or the crystallinity of the core [25]. In other words, the effect of introducing oxygen and nitrogen is to generate states within the carbon energy gap, with nitrogen states being shallower than oxygen states and therefore requiring less energy for transitions. These functional groups act as emitting centers or as modifiers of electronic levels, with a tendency for maximum emission towards longer wavelengths observed in CDs with higher nitrogen content, which is associated with a higher ripeness of the orange peels.

### 3.6. Morphology and Particle Size

**Figure 6:**
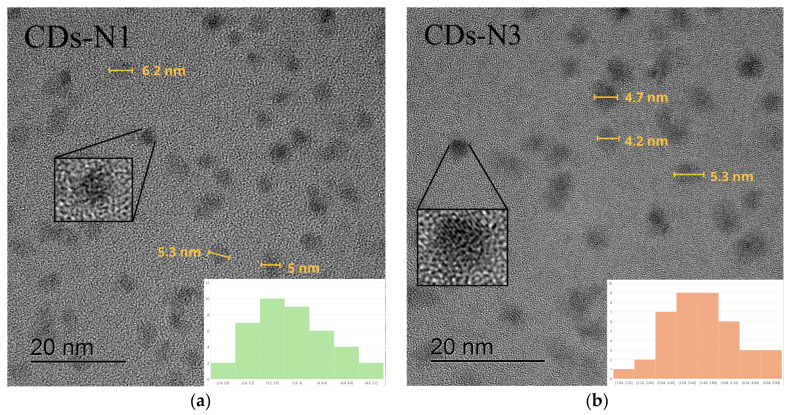
TEM images of carbon dots obtained from orange peels at different ripeness stages: (**a**) CDs-N1 and (**b**) CDs-N3. The insets show the size distribution of the carbon dots obtained from the analysis of a sample of 40 particles.

ImageJ software (v1.53) was used to analyze the size distribution. In the high-magnification images of the CDs (Figure 6a,b), sizes smaller than 10 nm are shown for both ripe and unripe samples. The crystal lattice fringes of the CDs-N3 core are more clearly defined, which is consistent with the decrease in the defect band in the Raman spectra. The images of both samples show similar sizes, indicating that the emission mechanism and shift in the CDs are due to surface trap states rather than core graphitization level.

## 4. Conclusions

This study demonstrated that the ripeness degree of orange peels directly influences the structure, surface chemistry, and optical properties of hydrothermally synthesized carbon dots. Less-ripe peels generated CDs with a higher content of oxygenated groups, a greater defect density (more intense D band), and a more efficient hydrolysis process of the precursor. In contrast, intermediate and ripe peels showed a lower tendency toward carbonization, a slight improvement in sp^2^ ordering, and a red shift in emission, associated with the growth of aromatic domains and the increase in surface nitrogenous groups. The results confirm that the precursor composition modulates nucleation, graphitic core development, and surface functionalization of CDs, making ripeness a useful parameter for tuning their optical and structural properties. These findings suggest that by exploring synthesis conditions and the nature of the precursor, the composition of carbon dots can be targeted in future applications such as sensors, imaging, and functional materials derived from organic waste.

## Data Availability

Data is contained within the article.
